# Retracted: Diagnostic Discrepancies Between Intraoperative Frozen Section and Permanent Histopathological Diagnosis of Brain Tumors

**DOI:** 10.5146/tjpath.2026.01551-rn

**Published:** 2026-05-30

**Authors:** Maher Kurdi, Saleh Baeesa, Yazid Maghrabi, Anas Bardeesi, Rothaina Saeedi, Taghreed Al-Sinani, Alaa Samkari, Ahmed Lary, Sahar Hakamy

**Affiliations:** Department of Pathology, King Abdulaziz University, Faculty of Medicine in Rabigh, Rabigh, Kingdom of Saudi Arabia; Division of Neurosurgery, King Abdulaziz University, Faculty of Medicine, Jeddah, Kingdom of Saudi Arabia; Department of Neuroscience, King Faisal Specialist Hospital and Research Center, Jeddah, Kingdom of Saudi Arabia; Department of Neuroscience, King Faisal Specialist Hospital and Research Center, Jeddah, Kingdom of Saudi Arabia; Department of Surgery, Division of Neurosurgery, King Abdulaziz University Hospital, Jeddah, Kingdom of Saudi Arabia; Department of Surgery, Division of Neurosurgery, King Fahad General Hospital, Jeddah, Kingdom of Saudi Arabia; Department of Pathology and Laboratory Medicine, King Saud Bin Abdulaziz University for Health Science, Jeddah, Kingdom of Saudi Arabia; Department of Surgery, Division of Neurosurgery, King Abdulaziz Medical City, Jeddah, Kingdom of Saudi Arabia; Center of Excellence in Genomic Medicine Research, King Abdulaziz University, Jeddah, Kingdom of Saudi Arabia

**Notice:** Prof. Dr. Volkan Adsay, Editor-in-Chief of the Turkish Journal of Pathology, is retracting the article titled “Diagnostic Discrepancies between Intraoperative Cytological Frozen Section and Permanent Histopathological Diagnosis of Brain Tumors”.

**Retracted Article:** Maher KURDI, Saleh BAEESA, Yazid MAGHRABI, Anas BARDEESI, Rothaina SAEEDI, Taghreed AL-SINANI, Alaa SAMKARI, Ahmed LARY, Sahar HAKAMY. Turkish Journal of Pathology 2022, Vol 38, No 1, pp. 034-039. DOI: 10.5146/tjpath.2021.01551.

**Reason for Retraction:** This retraction is issued regarding redundant (duplicate) publication. Following an editorial investigation, it was determined that the findings and substantial content of this article were previously published in the Journal of Cytology & Histology (DOI: 10.37421/2157-7099.2021.12.585) without proper attribution, disclosure, or justification provided to the editors.

**Purpose:** The purpose of this retraction is to correct the scientific record and ensure the integrity of the literature; it is not intended to punish the authors. The Editorial Board of the Turkish Journal of Pathology apologizes to the scientific community for any confusion caused by this redundant publication.

